# Micropapillary bladder cancer: a review of Léon Bérard Cancer Center experience

**DOI:** 10.1186/1471-2490-9-5

**Published:** 2009-06-17

**Authors:** Pierre Heudel, Fadi El Karak, Nabil Ismaili, Jean-Pierre Droz, Aude Flechon

**Affiliations:** 1 Léon Bérard Cancer Center, 28 Rue Laennec, 69008 Lyon, France

## Abstract

**Background:**

Micropapillary bladder cancer is a rare and aggressive variant of urothelial carcinoma. A retrospective review of our experience in management of patients with muscle-invasive or metastatic micropapillary bladder cancer was performed to better define the behavior of this disease.

**Methods:**

We reviewed the records of the 11 patients with micropapillary bladder cancer who were evaluated and treated at Léon Bérard Cancer Center between 1994 and 2007, accounting for 1,2% of all urothelial tumors treated in this institution.

**Results:**

Mean patients age was 60 years. The majority of patients (72%) were diagnosed after 2004. After a median follow-up of 31.7 months, median overall survival was 19 months. Two patients presented with stage II, one with stage III and eight with stage IV disease All 5 patients who had node positive metastases and treated with radical surgery and adjuvant chemotherapy relapsed and had a disease free survival of 9.6 months.

**Conclusion:**

Micropapillary bladder cancer is probably an underreported variant of urothelial carcinoma associated with poor prognosis. Adjuvant chemotherapy might have a questionable efficacy and the optimal treatment strategy is yet to be defined.

## Background

Micropapillary bladder carcinoma is a rare variant of urothelial bladder cancer recently described by Amin *et al *[1], and accounting for 0,7 to 2,2% of all urothelial tumors. It is associated with a rapid growth rate and an advanced stage at presentation [1,2]. We retrospectively analyzed 11 consecutive cases of muscle-invasive or metastatic micropapillary bladder cancer treated at the Léon Bérard cancer center between 1994 and 2007 in order to better define the behaviour of this aggressive disease.

## Methods

We retrospectively searched the files of all patients with muscle-invasive or metastatic micropapillary bladder carcinoma treated at the Léon Bérard cancer center between 1994 and 2007. Patients were considered to have micropapillary disease if the pathology report revealed any micropapillary component (MPC) in their tumor. Patient medical records were analyzed for demographic characteristics, clinical stage and outcome. Radiology, pathology and surgical reports were reviewed to determine the pathological staging at the time of cystectomy using the 1997 TNM classification for genitourinary tumors [3]. Chemotherapy regimens, radiotherapy doses and surgical modality were also recorded. Overall survival was calculated from the date of diagnosis to the date of death or the date of last follow-up. Estimation of survival curves was performed using the Kaplan-Meier technique.

## Results

### Characteristics of patients at initial presentation

Between 1994 and 2007, 911 patients with urothelial carcinoma were treated at the Léon Bérard cancer Center. Eleven patients presented with muscle-invasive or metastatic disease and had micropapillary features. Median age at diagnosis was 60 years (48 to 76 years) and male/female ratio was 9/1. Eighty percent of patients had previous smoking history. All patients had grade 3 urothelial carcinoma and 35% had an associated in situ carcinoma. One patient was diagnosed before year 2000, 1 in 2001, 1 in 2002 and 8 between 2004 and 2007. Two patients presented with stage II, one with stage III and eight with stage IV disease. The average time between initial symptoms and the beginning of treatment is 3,8 months, while this period is 2 months for patients treated with surgery initially.

### Treatment and outcome of patients with muscle invasive non metastatic disease

One patient had a cystoprostatectomy and pelvic lymph node dissection. He was alive with no evidence of disease recurrence two years after the diagnosis. The second patient received concomitant pelvic chemoradiation at the dose of 60 Grays. He relapsed 23 month later, received subsequently two chemotherapy regimens and died ten months later. The third patient refused treatment and died 8 months later.

### Treatment and outcome of patients with metastatic disease

Seven patients had only regional lymph node metastases while one had distant metastases. Among those seven patients, six had cystoprostatectomy and pelvic lymph nodes dissection of whom, five received 4 cycles of adjuvant MVAC chemotherapy (methotrexate, vinblastin, adriamycin and cisplatin). All of them relapsed and died from metastases. One patient received four cycles of MVAC chemotherapy and achieved a clinical complete response. He had pelvic irradiation at the dose of 65 Grays but died ten month later from disease progression. The patient who presented with distant metastases was treated with chemotherapy and died ten months after the initial diagnosis.

Table S1 [see additional file 1] summarizes the initial management of patients with muscle invasive or metastatic micropapillary bladder cancer and table S2 [see additional file 2] summarizes different chemotherapeutic regimens used.

### Metastatic sites

Ten out of the 11 patients developed distant metastases during the follow up. The most frequent solid metastatic site was the lung followed by liver and bone (Table S3 [see additional file 3]).

### Survival data

**Figure 1:**
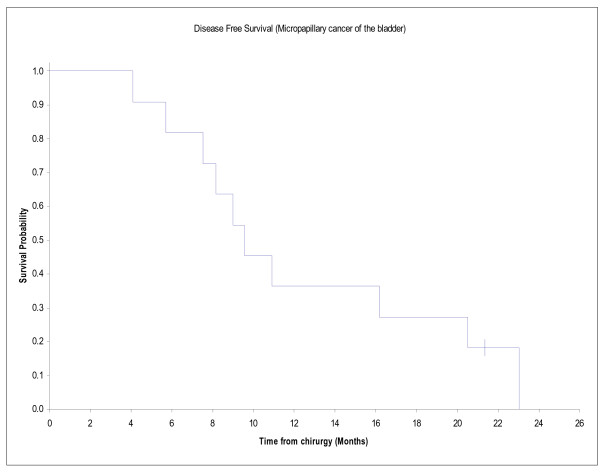
**Disease free survival of 11 patients with micropapillary muscle invasive or metastatic bladder cancer**. Represents the Disease free survival of 11 patients with micropapillary muscle invasive or metastatic bladder cancer.

**Figure 2:**
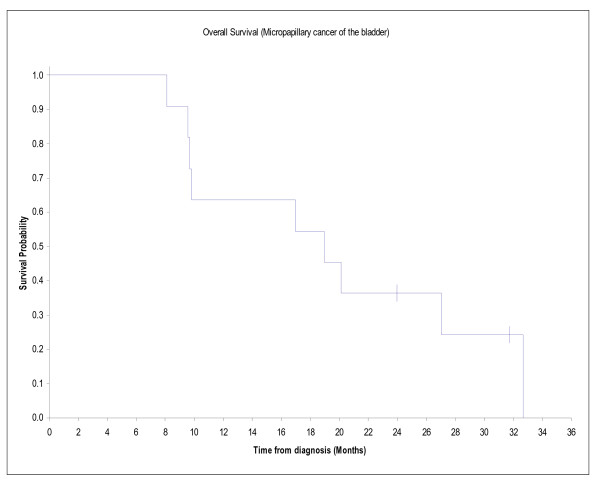
**Overall survival of 11 patients with micropapillary muscle invasive or metastatic bladder cancer**. Description: represents the Overall survival of 11 patients with micropapillary muscle invasive or metastatic bladder cancer.

At a median follow up of 31.7 months, 9 patients have died. Disease free survival was 9,6 months. One and two-years survival were 64% and 36% respectively and median survival was 19 months. Progression free survival is illustrated in figure 1 and overall survival in figure 2.

## Discussion

Carcinoma with MPC was first described in 1982 as a variant of endometrial carcinoma initially designed as papillary serous carcinoma of the uterus [4]. Later, this subtype was depicted in thyroid [5], breast [6], urinary tract [1] and lung cancers [7].

Amin *et al *were the first to describe this entity in urothelial carcinoma, in 1994. They identified two distinct morphologic appearances. On the surface MPC are characterized by small papillary tufts and slender, delicate processes, often with a central fibrovascular core while in the invasive part, the cells form small tight nests or balls often seen within retracted connective tissue spaces [1]. Associated typical urothelial carcinoma is usually high grade as was shown in our series. Immunohistochemical profile typically shows CK7+/CK20+(CA125+/-) suggesting that it is a form of glandular differentiation in urothelial carcinoma [8,9].

Described less than 15 years ago, micropapillary bladder cancer is likely underreported. In our institution, only 1 patient was diagnosed before year 2000 while the other 10 patients were identified between 2001 and 2007 mainly after 2004 (8 cases). This suggests that the reported frequency of micropapillary bladder cancer might increase in the future as pathologists and oncologists become more aware of its description and particular clinical behavior.

To date, there are no prospective trials in micropapillary bladder cancer. Our retrospective study is the third largest clinical series of muscle invasive and metastatic bladder cancer reported in the literature after Kamat *et al *who described 56 consecutive cases [10] and Johansson *et a*l who reported 17 consecutive cases [2]. The other relatively large series are clinicopathological studies with no correlation between treatment and outcome [1,8,11].

The presence of MPC in urothelial carcinoma was found to be associated with an advanced stage of disease at the time of presentation and an aggressive clinical course [8,9,11,12]. In 2004, Samaratunga *et al*. [8] reported 20 cases of micropapillary bladder cancer and correlated the pathological stage and prognosis with the extent and extension of the micropapillary component on the pathology specimen. In patients with superficial bladder cancer, intravesical BCG was shown to be ineffective and the authors concluded that the optimal treatment strategy for non-invasive bladder cancer with micropapillary components should be a radical cystectomy [13]. The same authors stated in another study that in muscle invasive surgically resectable disease, radical cystectomy should be performed precociously. In this paper, the incidence of pathologic upstaging was 52.7% and the incidence of occult lymph node disease detected at the time of cystectomy was 27.3%. Moreover patients undergoing neoadjuvant chemotherapy were found to have a non-organ-confined disease (≥ T3) in 68% of cases compared to patients treated with upfront surgery (34%) (p = 0,0157). There was no survival benefit from the addition of neoadjuvant chemotherapy and the authors recommended no such approach especially in the absence of lymphovascular invasion [10]. Moreover, in this study, of the 15 patients with lymph node positive disease, 11 received adjuvant chemotherapy after radical cystectomy, and only 5 were alive at the time of last follow-up (follow-up time ranging from 29 to 42 months). This tends to show the chemoresistant characters of micropapillary bladder cancer. In fact, in other organ sites, micropapillary carcinoma appears to be less responsive to chemotherapy. For example, a retrospective study evaluated the pattern of chemoresistance in invasive micropapillary/low grade serous ovarian carcinoma and high grade serous ovarian carcinoma. Authors concluded that patients with recurrent invasive micropapillary/low grade serous ovarian carcinoma were more likely to manifest drug resistance to standart chemotherapy agents (platinum and paclitaxel) [14].

The present study showed occurrence of the disease in relatively young patients (median age was 60 years) and an associated poor prognosis. Patients who received multimodal treatment had a median disease free survival of 11 months and an overall survival of 21 months. All 5 patients with node positive disease who received surgery followed by adjuvant MVAC regimen relapsed within a median follow up of 20,2 months. This is in sharp contrast with other studies that used same treatment modalities for node positive resected urothelial carcinoma [15-18]. In the study by Stokle *et al*, only 3 among 11 patients relapsed after a median follow-up period of 13.5 months [15]. In the study by Freiha *et al*, 10 patients out of 18 relapsed after a median follow-up of 62 months [16]. This further emphasizes on the aggressive character and chemoresistance of this variant of urothelial carcinoma.

## Conclusion

Micropapillary bladder cancer is very likely an underreported variant of urothelial carcinoma. It is associated with a poor prognosis. The low incidence of this variant renders the conduct of randomized trials rather impossible and drawing clear guidelines for its management is subsequently difficult. Nevertheless, our series suggests that adjuvant chemotherapy might have a questionable efficacy especially in node positive disease, and the optimal treatment modality remains to define.

## Competing interests

The authors declare that they have no competing interests.

## Authors' contributions

PH participed in acquisition of data, analysis and interpretation of data, wrote the article. FEL participed in acquisition of data, analysis and interpretation of data. NI participed in acquisition of data. JPD participed in analysis and interpretation of data, revising manuscript. AF participed in analysis and interpretation of data, revising manuscript. All authors read and approved the final manuscript.

## Pre-publication history

The pre-publication history for this paper can be accessed here:



## Supplementary Material

Additional file 1 **Table S1. Initial management of patients with micropapillary bladder cancer by stage**. represents the Initial management of patients with micropapillary bladder cancer by stage. Click here for file

Additional file 2 **Table S2. Chemotherapeutic regimens**. represent chemotherapeutic regimens. Click here for file

Additional file 3 **Table S3. Metastatic sites**. represents metastatic sites. Click here for file

## References

[B1] Amin MB, Ro JY, El-Sharkawy T (1994). Micropapillary variant of transitional cell carcinoma of the urinary bladder. Histologic pattern resembling ovarian papillary serous carcinoma. Am J Surg Pathol.

[B2] Johansson SL, Borghede G, Holmäng S (1999). Micropapillary bladder carcinoma: a clinicopathological study of 20 cases. J Urol.

[B3] Sobin LH, Wittekind Ch, Sobin LH, Wittekind Ch (1997). TNM classification of tumours of the urinary bladder. TNM classification of malignant tumors.

[B4] Hendrickson M, Ross J, Eifel P (1982). Uterine papillary serous carcinoma: a highly malignant form of endometrial adenocarcinoma. Am J Surg Pathol.

[B5] McDougall IR, Camargo CA (2007). Treatment of Micropapillary Carcinoma of the Thyroid: Where Do We Draw the Line?. Thyroid.

[B6] Siriaunkgul S, Tavassoli FA (1993). Invasive micropapillary carcinoma of the breast. Mod Pathol.

[B7] Amin MB, Tamboli P, Merchant SH (2002). Micropapillary component in lung adenocarcinoma: a distinctive histologic feature with possible prognostic significance. Am J Surg Pathol.

[B8] Samaratunga H, Khoo K (2004). Micropapillary variant of urothelial carcinoma of the urinary bladder; a clinicopathological and immunohistochemical study. Histopathology.

[B9] Kuroda N, Tamura M, Ohara M (2006). Invasive micropapillary carcinoma of the urinary bladder: an immunohistochemical study of neoplastic and stromal cells. Int J Urol.

[B10] Kamat AM, Dinney CP, Gee JR (2007). Micropapillary bladder cancer: a review of the University of Texas M.D. Anderson Cancer Center experience with 100 consecutive patients. Cancer.

[B11] Alvarado-Cabrero I, Sierra-Santiesteban FI, Mantilla-Morales A (2005). Micropapillary carcinoma of the urothelial tract. A clinicopathologic study of 38 cases. Ann Diagn Pathol.

[B12] Nishizawa K, Kobayashi T, Mitsumori K (2005). Micropapillary bladder cancer. Int J Urol.

[B13] Kamat AM, Gee JR, Dinney CP (2006). The case for early cystectomy in the treatment of nonmuscle invasive micropapillary bladder carcinoma. J Urol.

[B14] Santillan A, Kim YW, Zahurak ML (2007). Differences of chemoresistance assay between invasive micropapillary/low grade serous ovarian carcinoma and high grade serous ovarian carcinoma. Int J Gynecol Cancer.

[B15] Stokle M, Meyenburg W, Wellek S (1992). Advanced bladder cancer (stages pT3b, pT4a, pN1 and pN2): improved survival after radical cystectomy and 3 adjuvant cycles of chemotherapy. Results of a controlled prospective study. J Urol.

[B16] Freiha F, Reese J, Torti FM (1996). A randomized trial of radical cystectomy versus radical cystectomy plus cisplatin, vinblastine and methotrexate chemotherapy for muscle invasive bladder cancer. J Urol.

[B17] Skinner DG, Daniels JR, Russel CA (1991). The role of adjuvant chemotherapy following cystectomy for invasive bladder cancer: a prospective comparative trial. J Urol.

[B18] Logothetis CJ, Johnson DE, Chong C (1988). Adjuvant cyclophosphamide, doxorubicin and cisplatin chemotherapy for bladder cancer: an update. J Clin Oncol.

