# Supplementary material for: Micropapillary bladder cancer: a review of Léon Bérard Cancer Center experience
Source: BMC Urol. 2009 Jun 17;9:5. doi: 10.1186/1471-2490-9-5 (PMC2713271; doi:10.1186/1471-2490-9-5)
Supplement: Additional file 1 — Table S1. Initial management of patients with micropapillary bladder cancer by stage. represents the Initial management of patients with micropapillary bladder cancer by stage. [file 1471-2490-9-5-S1.pdf]

Stage and initial management	All patients (11)	recurrence or metastases (10)
**Locally advanced disease**	3	2
radical cystoprostatectomy (pT2 pN0 M0)	1	0
no treatment (T2N0M0)	1	1
Radiochemotherapy (T4N0M0)	1	1
**Pelvic lymph nodes involvement**	7	7
radical cystectomy and adjuvant chemotherapy	5	5
radical cystectomy alone	1	1
chemotherapy and radiochemotherpy concomitant	1	1
**Distant metastatic disease**	1	1
chemotherapy alone	1	1

Table 1. Initial management of patients with micropapillary bladder cancer by stage
