# Supplementary material for: Micropapillary bladder cancer: a review of Léon Bérard Cancer Center experience
Source: BMC Urol. 2009 Jun 17;9:5. doi: 10.1186/1471-2490-9-5 (PMC2713271; doi:10.1186/1471-2490-9-5)
Supplement: Additional file 2 — Table S2. Chemotherapeutic regimens. represent chemotherapeutic regimens. [file 1471-2490-9-5-S2.pdf]

chemotherapeutics agents	adjuvant chemotherapy	sequential radiochemotherapy	concomitant radiochemotherapy	first line metastatic	second line metastatic
Cisplatine, methotrexate, doxorubicine and vinblastine	5	1		2	
Cisplatine and gemcitabine				2	
Carboplatine and gemcitabine				1	
Gemcitabine				1	1
Navelbine					1
Cisplatine			1		

Table 2. Chemotherapeutics regimens
