# Supplementary material for: Micropapillary bladder cancer: a review of Léon Bérard Cancer Center experience
Source: BMC Urol. 2009 Jun 17;9:5. doi: 10.1186/1471-2490-9-5 (PMC2713271; doi:10.1186/1471-2490-9-5)
Supplement: Additional file 3 — Table S3. Metastatic sites. represents metastatic sites. [file 1471-2490-9-5-S3.pdf]

Metastatic site	No Pts (%)
Retroperitoneal or pelvic lymph nodes	6 (33)
lung	5 (27)
liver	3 (17)
bone	3 (17)
brain	1 (6)

Table 3. Metastatic sites
